# Enhancing Intrusion Detection in Wireless Sensor Networks Using a GSWO-CatBoost Approach

**DOI:** 10.3390/s24113339

**Published:** 2024-05-23

**Authors:** Thuan Minh Nguyen, Hanh Hong-Phuc Vo, Myungsik Yoo

**Affiliations:** 1 Department of Electronic Engineering, Soongsil University, Seoul 06978, Republic of Korea; 1102360003@soongsil.ac.kr (T.M.N.); hanhvo@soongsil.ac.kr (H.H.-P.V.); 2 School of Electronic Engineering, Soongsil University, Seoul 06978, Republic of Korea

**Keywords:** wireless sensor networks, intrusion detection, feature selection, hyperparameter optimization, machine learning

## Abstract

Intrusion detection systems (IDSs) in wireless sensor networks (WSNs) rely heavily on effective feature selection (FS) for enhanced efficacy. This study proposes a novel approach called Genetic Sacrificial Whale Optimization (GSWO) to address the limitations of conventional methods. GSWO combines a genetic algorithm (GA) and whale optimization algorithms (WOA) modified by applying a new three-population division strategy with a proposed conditional inherited choice (CIC) to overcome premature convergence in WOA. The proposed approach achieves a balance between exploration and exploitation and enhances global search abilities. Additionally, the CatBoost model is employed for classification, effectively handling categorical data with complex patterns. A new technique for fine-tuning CatBoost’s hyperparameters is introduced, using effective quantization and the GSWO strategy. Extensive experimentation on various datasets demonstrates the superiority of GSWO-CatBoost, achieving higher accuracy rates on the WSN-DS, WSNBFSF, NSL-KDD, and CICIDS2017 datasets than the existing approaches. The comprehensive evaluations highlight the real-time applicability and accuracy of the proposed method across diverse data sources, including specialized WSN datasets and established benchmarks. Specifically, our GSWO-CatBoost method has an inference time nearly 100 times faster than deep learning methods while achieving high accuracy rates of 99.65%, 99.99%, 99.76%, and 99.74% for WSN-DS, WSNBFSF, NSL-KDD, and CICIDS2017, respectively.

## 1. Introduction

Wireless sensor networks (WSNs) have emerged as a pivotal technology with a growing presence in various application areas, including environmental monitoring, healthcare, industrial automation, and smart cities [1]. These networks of tiny, interconnected sensors are ideally suited for collecting real-time information about their surroundings. In recent years, there has been a notable upsurge in WSN research, driven by the extensive array of real-time applications [2]. In particular, a noteworthy study published in IEEE Transactions on Wireless Communications (TWC) in 2023 focused on the throughput maximization of wireless-powered communication networks with mobile access points [3]. In addition to the aforementioned study, numerous recent works have contributed to the advancement of WSNs. For example, “Energy-Saving Routing Protocols for Smart Cities” (Medeiros et al. [4], 2022) proposed energy-efficient routing protocols tailored for WSNs deployed in smart cities. Furthermore, “Secure and scalable data aggregation techniques for healthcare monitoring in WSN” (Vidyapeeth et al. [5], 2023) addressed the critical aspect of data security in WSNs deployed in healthcare settings. These recent works demonstrate the ongoing efforts of researchers to tackle various challenges in WSNs, including throughput maximization, energy efficiency, and data security.

Within the context of security, the reliability of WSNs is seriously threatened by numerous typical attacks, including Hello Flooding, Wormholes, Sinkholes, and Jamming, which cause abnormal traffic behavior by upsetting the network’s traffic patterns [6]. Therefore, intrusion detection in WSNs stands out as a crucial topic [7]. Because intrusion detection addresses the critical issue of ensuring data integrity and network security in environments with constrained resources, its importance cannot be understated. Effective identification and mitigation of such attacks are necessary to preserve the integrity and functionality of these networks. The challenges of intrusion detection posed by WSNs are fundamentally different from those encountered by conventional networks [8].

It has been established that the use of machine learning (ML) is reliable for network intrusion detection [9,10]. In addition, ML has recently emerged as a promising method for handling intricate data [11,12]. Furthermore, its extensive utilization in identifying anomalous network activities may be attributed to its ease of use and effectiveness [13,14,15]. However, the increasing network traffic poses challenges to the performance of ML in real-time data analysis because excessive irrelevant data are being gathered.

In general, the ultimate efficacy of ML depends on the quality of the available data [16], the relevance of features, and the extent to which models and algorithms can approach their performance ceiling. As computer technology continues to find applications in various facets of human society, an increasing number of real-world dataset feature spaces encompass tens of thousands of dimensions [17]. Nonetheless, only a fraction of these dimensions truly encapsulate the intrinsic nature of the phenomena under investigation. This subset of pertinent features is overshadowed by the profusion of irrelevant and redundant features, which severely impairs the performance of ML algorithms.

To address this challenge, numerous methodologies have been proposed to leverage ML with population-based algorithms inspired by natural processes. Researchers have focused their investigations on several prominent metaheuristic algorithms whose components can be optimized, and those binary counterparts are appropriately tailored to address the intricacies of FS. Among these, the whale optimization algorithm (WOA) [18] has emerged as a noteworthy candidate owing to its adaptable characteristics, minimal control parameters, and straightforward structural design. However, the applicability of the WOA and its variants is constrained when addressing high-dimensional, intricate problems. Notably, issues such as diminished population diversity and premature convergence to suboptimal solutions have become more pronounced, as evidenced by empirical assessments and analytical findings [19]. Consequently, our study is motivated by the imperative to propose enhancements to WOA that facilitate the discovery of superior solutions, striking a harmonious balance between extracting a feature subset of elevated quality to augment prediction accuracy and achieving dimensional reduction to mitigate inference time.

Furthermore, tuning the hyperparameters of ML models is critical for optimizing the performance and predictive accuracy of these models [20]. This process involves adjusting the hyperparameters and model configurations to ensure the most effective adaptation to the resource-constrained and dynamically changing nature of WSNs.

In this study, we introduce a specifically designed intrusion detection model, assisted by metaheuristic optimization, to comply with WSN characteristics. We briefly summarize our contributions as follows:

- We propose a new approach called Genetic Sacrificial Whale Optimization (GSWO) that ingeniously combines a genetic algorithm (GA) and WOA modified by applying a new three-population division strategy with the proposed conditional inherited choice for FS. The proposed algorithm can eliminate the premature convergence of the standard WOA and strike a balance between exploration and exploitation abilities.
- Moreover, we harnessed the capabilities of the CatBoost model as a classifier, distinguishing between the benign data and diverse attack patterns within the dataset.
- In addition, we introduce a new method for fine-tuning CatBoost’s hyperparameters, incorporating quantization and an optimization approach akin to FS (GSWO).
- Finally, we rigorously evaluate the proposed methodology using a comprehensive range of datasets, encompassing established benchmarks such as CICIDS 2017 and NSL-KDD as well as specialized WSN datasets including the WSN dataset and the WSNBFSF dataset, which was published in 2023. These comprehensive evaluations underscore the accuracy and real-time applicability of the proposed method on various data sources.

The subsequent sections of this manuscript are structured as follows: Section 2 delves into the related literature and research. Section 3 explains the comprehensive IDS proposed for WSNs. Section 4 expounds on the GSWO algorithm, detailing its application in FS and the fine-tuning of hyperparameters. The experimental results and performance analysis are presented in Section 5. Finally, Section 6 provides the concluding remarks and outlines avenues for future research.

## 2. Related Work

### 2.1. WSN Intrusion Detection

Almomani et al. [21] developed a specialized dataset called WSN-DS, tailored for WSN intrusion detection. They employed an artificial neural network (ANN) on this dataset, which resulted in enhanced accuracy in the detection and classification of DoS attacks. Subsequently, in 2023, Dener et al. [22] introduced a novel dataset, WSN-BFSF, specifically designed to detect DoS attacks in WSNs. Their investigation involved the evaluation of four ML and eight deep-learning models, yielding notable outcomes.

Vinayakumar et al. [23] devised a hybrid IDS for detecting and classifying attacks. They introduced a scalable deep neural network (DNN) framework called scale-hybrid-IDS-AlertNet, which was designed to combat network attacks. The proposed methodology assessed diverse datasets, including CICIDS2017, WSN-DS, UNSW-NB15, and NSL-KDD, and exhibited commendable accuracy across various network traffic types. However, their study lacked a comprehensive discussion regarding computational time efficiency [24].

Le et al. [6] implemented a random forest algorithm to classify four types of DoS attacks using the WSN-DS dataset. This study involved a comparative analysis of the performance of random forest and ANN algorithms. The findings demonstrated the efficacy of random forest as a robust machine-learning method adept at overcoming overfitting challenges and outperforming ANN. However, it is pertinent to note that the study’s results were derived from a relatively limited dataset comprising 94,042 instances during the testing phase [25]. It is worth mentioning that this investigation exclusively considered the LEACH routing protocol, while other protocols remained unexplored.

Salmi and Oughdir [26] developed and implemented various models, including DNN, convolutional neural networks (CNNs), recurrent neural networks (RNNs), and hybrid RNN–CNN architectures, for the detection of DoS attacks in WSNs. The models were trained using the WSN-DS dataset, with the CNN exhibiting superior performance with an accuracy rate of 98.79%. Notably, compared to ML models, deep learning models introduce a heightened computational overhead. Given the inherent constraints of WSNs, lightweight security solutions are imperative for addressing these network limitations.

Although there have been significant advancements in the development of specialized datasets and the application of various machine learning and deep learning models for intrusion detection in Wireless Sensor Networks (WSNs), there is still a significant research gap in terms of accuracy and inference time. This shortcoming stems from the intrinsic complexity of the current models, which are applied directly to raw data with high noise levels and no FS stage, leading to long inference times in real-world applications. Moreover, the aforementioned ML models do not yet incorporate hyperparameter optimization strategies designed to obtain the best possible performance of the models in certain wireless sensor network situations.

### 2.2. Metaheuristic Optimization Inspired Feature Selection for Intrusion Detection

Jiang et al. [27] introduced SLGBM, an IDS tailored for WSNs. This study integrated the Sequence Backward Feature Selection (SBS) algorithm with the LightGBM classification algorithm to enhance the detection rate while mitigating computational overhead. Through experimentation utilizing the WSN-DS dataset, the SLGBM method demonstrated superior performance in terms of the F1-score for the four types of network attacks compared with existing detection methods. However, this study did not explicitly address the scalability of the SLGBM method.

Liu et al. [28] proposed a particle swarm optimization-based gradient descent (PSO-LightGBM) model for intrusion detection in IoT, which aimed to address the limitations of traditional intrusion detection technology in a complex and changeable IoT environment. The model uses PSO-LightGBM to extract features from the data and input them into a one-class SVM (OCSVM) for the discovery and identification of malicious data. The proposed model performs well in terms of accuracy and false alarm rate (FAR) and shows good robustness and reliability on the UNSW-NB15 dataset. However, there have been no comparisons in terms of effectiveness between PSO and other metaheuristic algorithms.

Vijayanand et al. [29] presented a novel FS approach combining WOA and genetic operators sequentially to enhance the accuracy of IDSs within Wireless Mesh Networks (WMN) utilizing Support Vector Machines (SVM). This study replaced the probing component of WOA with crossover and mutation operators. A performance evaluation conducted on the CICIDS2017 and ADFA-LD standard datasets showed the method’s superiority over conventional WOA and GA in terms of attack detection rate, accuracy, and suitability. However, this study did not provide insights into the efficiency of the proposed method compared to other metaheuristic algorithms.

Hussain et al. [30] proposed an IDS designed specifically for WSNs, utilizing a CNN architecture for classification and a hybrid WOA–artificial bee colony (ABC) algorithm for FS. When combined with the recommended CNN architecture, this method outperformed PSO and achieved an overall accuracy of 98%, according to the experimental evaluation on the NSL-KDD dataset. However, it is important to mention that the hybrid WOA-ABC algorithm, which combines two large optimization algorithms, might increase the computing complexity and resource requirement.

Mohiuddin et al. [31] presented an effective IDS that combines a whale optimization technique, which is a modified wrapper-based algorithm with a sine-cosine FS algorithm and a Weighted Extreme Gradient Boosting (XgBoost) classifier. The objective of this method is to enhance the prediction quality and prevent local optima by broadening the search and effectively selecting an ideal solution using the sine–cosine function. Using the UNSW-NB15 and CICIDS datasets, the proposed model successfully classified binary and multi-class attacks with notable accuracy, precision, recall, and F1-score metrics. Nevertheless, significant constraints exist concerning the scalability, computational efficiency, and applicability of the proposed model to various network contexts that have not been discussed.

Kasongo et al. [32] proposed a method that employs a GA for FS and a random forest (RF) model for classification within an IDS designed for Industrial Internet of Things (IIoT) networks. The GA-RF approach attained a test accuracy of 87.61% and an Area Under the Curve (AUC) of 0.98 for binary classification, demonstrating superior performance compared with existing IDS frameworks. The effectiveness and robustness of the proposed approach are evaluated using the UNSW-NB15 dataset. This methodology integrates the advantages of GA for FS and RF for classification, thereby enhancing the security, privacy, and integrity of IIoT networks.

A thorough analysis of the aforementioned studies on WSN intrusion detection specifically concentrates on FS inspired by metaheuristic optimization, with a particular emphasis on the WOA, revealing significant trends and unmet needs. First, more research is needed to determine whether these metaheuristic optimization-based FS techniques, particularly WOA, are applicable and scalable in a variety of large-scale network contexts. Secondly, there is a lack of discussion on how to effectively compare alternative metaheuristic optimization techniques for FS over a range of intrusion detection datasets. Finally, we acknowledge that a close examination of the tradeoff between the WOA’s exploration and exploitation capabilities is necessary to propose a novel approach for improving WOA for FS.

### 2.3. Fine-Tuning Hyperparameters for Machine Learning Model

Random search, which was developed by Bergstra et al. [33], is a traditional method that uses stochastic selection to determine hyperparameter values. Although a random search is known for being quick, it does not always guarantee the best possible results. Grid Search, on the other hand, provides an extensive examination of the hyperparameter space for a predetermined subset. It should be noted that using the Grid Search approach might become unfeasible if the parameter space is too large.

Bayesian optimization, introduced by Quitadamo et al. [34], is a robust methodology for fine-tuning the hyperparameters associated with computationally demanding and resource-intensive processes. It operates by generating a probabilistic model that is subsequently optimized to strategically guide the selection of the next evaluation points for the model’s objective function. All prior function evaluations inform this decision.

Furthermore, Olson et al. [35] proposed the Tree-based Pipeline Optimization Tool (TPOT), an algorithm that leverages genetic programming techniques, and demonstrated its capacity to autonomously identify high-performing machine learning algorithm pipelines without requiring domain-specific expertise or human intervention. Notably, the genetic approach is particularly well-suited for expansive search spaces, although it is essential to acknowledge that computational efficiency may be limited, albeit adjustable, through the manipulation of parameters such as the number of generations and population size.

SHERPA [36], introduced by Hertel et al., is an advanced hyperparameter tuning methodology primarily engineered to cater to tasks requiring resource-intensive and iterative function assessments, particularly in contexts such as hyperparameter optimization for deep learning models. Akiba et al. [37] proposed a new hyperparameter optimization software called Optuna (version 3.6.1), which is characterized by its capacity to dynamically construct parameter search spaces, efficiently implement search and pruning strategies, and offer a flexible and easily deployable architecture suitable for a wide range of applications.

An illustrative example is the work by Gabriel et al., who introduced a novel model termed BSOXGB [38] (BorutaShap feature selection combined with Optuna hyperparameter tuning of eXtremely Gradient Boost), achieving a notable accuracy of 97.70% in the diagnosis of Coronary Artery Disease (CAD). They underscored the importance of data preprocessing (DP) and hyperparameter tuning (HP) to ensure optimal performance in CAD prediction models.

In conclusion, based on the recent studies comprehensively reviewed above, we found that to achieve the highest accuracy and efficiency, it is necessary to combine and enhance pertinent FS, ML models, and hyperparameter optimization techniques. Therefore, we propose a novel IDS that integrates a proposed optimization strategy called GSWO to select pertinent features and fine-tune hyperparameters for the CatBoost classifier. A summary of the intrusion detection methods mentioned above and our work in this context is presented in Table 1.

## 3. Proposed System

**Figure 1:**
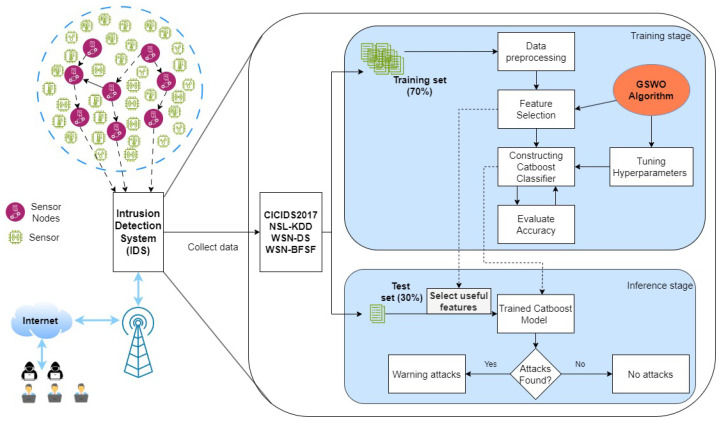
Overall intrusion detection system architecture.

An intrusion is defined as a malicious activity that gains network access and implements unauthorized tasks. IDSs’ role is to secure networks by detecting malicious and unauthorized activities. Figure 1 describes the proposed IDS in WSNs and the overall procedure inside it from the training stage to the testing stage.

The proposed intrusion detection model comprises two stages. The first stage involves selecting the most prominent features, and the second stage involves classification with hyperparameter optimization. After preprocessing the data to eliminate redundant data and noise, some informative features are selected using our proposed GSWO algorithm and the CatBoost classifier as fitness functions. A CatBoost-based ensemble learning approach [39,40] was developed to effectively detect and classify intrusive behaviors in terms of both accuracy and inference time when implemented in a real environment. However, few studies have used CatBoost in this field because of the difficulty of tuning the parameters to optimize the model and obtain the highest efficiency. The CatBoost hyperparameters affect the complexity of the model, training time, chance of overfitting, and speed of convergence. Therefore, the proposed GSWO algorithm was employed to address this problem after selecting the most informative features from the former block. After completing this specific phase in the training stage, the trained and optimized CatBoost model verifies its performance with the test set, including only useful features.

## 4. Proposed Work

### 4.1. Preliminaries

#### 4.1.1. Whale Optimization Algorithm

Mirjalili and Lewis drew inspiration from the predation behavior of humpback whales to formulate a WOA in 2016 [18]. The algorithm emulates the feeding strategy of humpback whales, which is characterized by diving near the surface of the water and employing a spiral pattern to trap prey within a net of bubbles. This predation behavior was delineated into three distinct stages.

- Encircling prey;
- Searching for prey;
- Bubble-net attacking.

The probability rate $\rho_{1}$ is employed for each whale to dynamically transition between the strategies of encircling prey/searching for prey and bubble-net attacks during the optimization process. In addition, individuals utilize a coefficient vector ${\vec{A}}^{t}$ to make strategic choices between the two techniques of encircling and searching for prey. The complete computation for updating the position of each individual in the WOA is determined by Equation (1). In this equation, the probability rate $\rho_{1}$ is a stochastic value within the range (0, 1), and the coefficient vectors ${\vec{A}}^{t}$ and ${\vec{C}}^{t}$ are calculated using Equations (2) and (3), respectively, where ${\vec{r}}_{a}$ and ${\vec{r}}_{c}$ are two vectors of a random number between [0,1], and vector *a* is a linear reduction from 2 to 0 in direct proportion to the number of iterations according to Equation (4), where t and M are the current iteration and the maximum number of allowed iterations. [·] is an element-by-element multiplication.

(1) $${\vec{X}}^{t+1}=\left\{\begin{array}{cc} Encircling & \left(\rho_{1}\leq 0.5\right)\;and\;\left(\left|{\vec{A}}^{t}\right|\leq 1\right).\;\\ Searching & \left(\rho_{1}\leq 0.5\right)\;and\;\left(\left|{\vec{A}}^{t}\right|>1\right).\;\\ Attacking & \left(\rho_{1}>0.5\right). \end{array}\right.$$

(2) $${\vec{A}}^{t}=2a\cdot r_{a}-a.$$

(3) $${\vec{C}}^{t}=2*{\vec{r}}_{c}.$$

(4) $$a=2-t\frac{2}{M}.$$

**Encircling prey technique:** The whales begin by encircling their target as a strategic move. Subsequently, they designated a search agent, making their choice dependent on the distance between each whale and the prey. Once the search agent was identified, all the whales within the collective adjusted their positions following the chosen leader. From a mathematical perspective, this process is expressed as follows:

(5) $${\vec{D}}^{t}=\left[{\vec{C}}^{t}\cdot {\vec{X}}_{*}^{t}-{\vec{X}}^{t}\right].$$

(6) $${\vec{X}}^{t+1}={\vec{X}}_{*}^{t}-{\vec{A}}^{t}\cdot {\vec{D}}^{t}.$$

Here, ${\vec{X}}_{*}^{t}$ is the position vector of the best whale (a locally optimal solution), while ${\vec{X}}^{t}$ is the position vector of the normal whale at the current iteration. ${\vec{X}}^{t+1}$ is the updated position vector of the same whale with ${\vec{X}}^{t}$. The distance between each whale and the search agent is expressed as ${\vec{D}}^{t}$.

**Searching for prey technique:** The WOA enhances the exploration capability across the whale population using the search-for-prey method. Exploration involves expanding the search space and identifying potential alternative solutions. In this method, a whale opts to advance toward a randomly chosen search agent position for global exploration, deviating from the conventional approach of moving toward the best-solution whale in the current iteration. Equations (7) and (8) are then employed to compute the random position of the search agent for updating, where ${\vec{X}}_{rand}^{t}$ is the position vector of the randomly chosen whale.

(7) $${\vec{D}}^{t}=\left[{\vec{C}}^{t}\cdot {\vec{X}}_{rand}^{t}-{\vec{X}}^{t}\right].$$

(8) $${\vec{X}}^{t+1}={\vec{X}}_{rand}^{t}-{\vec{A}}^{t}\cdot {\vec{D}}^{t}.$$

**Bubble-net attack technique:** The whale enhances its exploitation ability by modifying the shrink position within the helix-shaped movement toward the prey, as governed by Equation (9). Here, the parameter *b* represents a constant indicative of the logarithmic spiral’s shape, and *l* constitutes a random number within the specified range of [−1, 1]. The computation of the distance between the search agent (without multiplying with coefficient ${\vec{C}}^{t}$) and each whale $D^{\prime t}$ is determined by Equation (10)

(9) $${\vec{X}}^{t+1}={\vec{D}}^{\prime t}\cdot e^{bl}\cdot cos\left(2\pi l\right)+{\vec{X}}_{*}^{t}.$$

(10) $${\vec{D}}^{\prime t}=\left|{\vec{X}}_{*}^{t}-{\vec{X}}^{t}\right|.$$

Despite being a well-known optimization technique, the WOA has some drawbacks, including poor population diversity, early convergence, and an imbalance across search strategies. Consequently, several WOA variations have been proposed since its debut to address its shortcomings. This study aims to address these shortcomings by appropriately integrating a GA and the WOA.

#### 4.1.2. Genetic Algorithm

**Figure 2:**
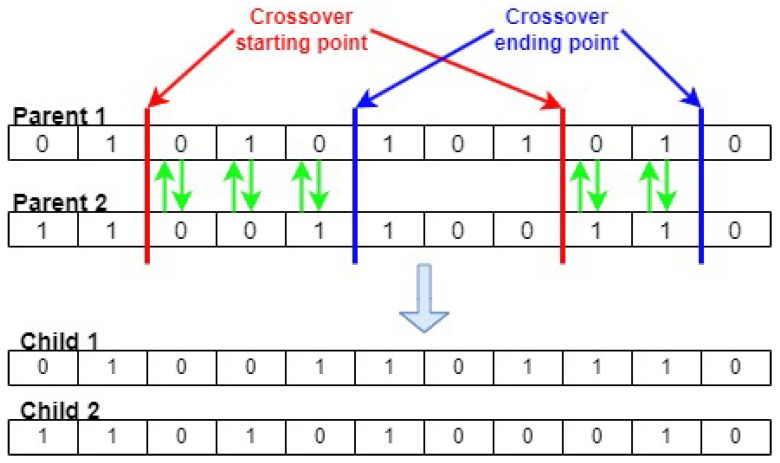
Crossover operator.

A GA is a heuristic technique used to improve results in a variety of applications. A previous study [41] shows how populations are represented as inputs in a chromosomal format and how they refine the parent population in each subsequent generation using three basic operations: selection, crossover, and mutation. The crossover process makes it easier for genetic features from the two parent organisms to combine. This is achieved by identifying a crossover spot on the parent chromosome structure. Following this, the last segment of one parent’s chromosome past the specified point is exchanged with the matching segment of the second parent’s chromosome and vice versa, creating a new population. The entire crossover process is illustrated in Figure 2 with the green arrows shows the process of exchanging the genes of two individuals. The offspring that are produced possess the traits of both parents. Compared to the parents’ chromosomes, the child’s unique combination may provide better or worse outcomes.

**Figure 3:**
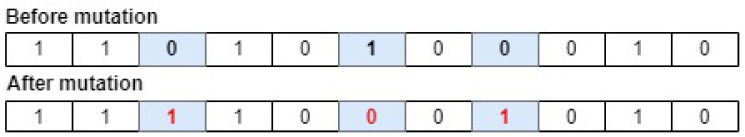
Mutation operator.

The purpose of the mutation operation is to avoid the problem of being trapped in local optima [42]. This process facilitated the generation of new individuals by modifying their random characteristics. Binary values are randomly reversed to convert 1s into 0s and vice versa in order to carry it out. For example, as graphically shown in Figure 3, the introduction of a mutation point at the individual’s third, sixth, and eighth positions cause the chromosome to mutate. The bold shows selected genes for mutation and the red color illustrates the new value of genes after mutation.

### 4.2. Proposed Genetic Sacrificial Whale Optimization

The GSWO proposed in this study was used to address global optimization and engineering design challenges. Based on fitness value, the whale population was divided into three subpopulations, each with a different number of individuals based on fitness values, as shown in Equations (11) and (12).

(11) $$v=Fit_{max}-Fit_{min},$$

where $Fit_{max}$ is the highest fitness value, while $Fit_{min}$ is the lowest fitness value in the population at a specific iteration. *v* is the distance between the best individual and the poorest individual.

(12) $$\left\{\begin{array}{c} Fit>Fit_{max}-\frac{1}{3}*v:Explore.\;\\ Fit<Fit_{min}+\frac{1}{3}*v:Exploit.\;\\ The\;remaining\;is\;hesitant\;subpopulation. \end{array}\right.$$

A corresponding calculation formula exists for the fitness value depending on the application. In this study, the fitness value is the false prediction rate of the model classification, so the smallest fitness value is the best.

The exploratory subpopulation includes whales with higher fitness values that are far from the current optimal solution. They are expected to strengthen the global exploration capabilities of the algorithm, allowing a systematic examination of more search spaces. Therefore, the searching for prey technique, which includes Equations (7) and (8), is suitable to execute the recalibration of this subpopulation’s location.

In contrast, individuals with lower fitness values, which fall into the exploitative subpopulation, need to increase inspection of the surrounding region to accelerate convergence [43]. Therefore, both the encircling prey technique and the bubble-net attack technique can be utilized randomly to modify the location of this subpopulation. For a complete description of the specifics, see Equation (13), where the probability rate $\rho_{2}$ is a random value between the interval [0,1]. The ${\vec{D}}^{t}$ and ${\vec{D}}^{\prime t}$ are given in Equations (5) and (10), respectively.

(13) $$\left\{\begin{array}{cc} {\vec{X}}^{t+1}={\vec{X}}_{*}^{t}-{\vec{A}}^{t}\cdot {\vec{D}}^{t} & (\rho_{2}>0.5).\\ {\vec{X}}^{t+1}={\vec{D}}^{\prime t}\cdot e^{bl}\cdot cos\left(2\pi l\right)+{\vec{X}}_{*}^{t} & (\rho_{2}\leq 0.5). \end{array}\right.$$

The hesitant subpopulation includes the remaining individuals with fitness values between good and poor. These individuals employ a balanced strategy that incorporates the whole process of traditional WOA and ensures a blend of exploration and exploitation. This strategy is represented mathematically by Equation (1).

**Figure 4:**
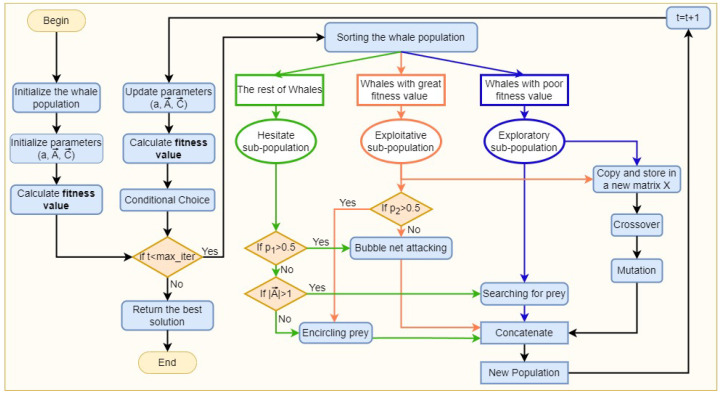
Genetic Sacrificial Whale Optimization Algorithm.

In GSWO, we re-evaluated all individuals in line with their current fitness levels after each iteration. Consequently, the makeup of the individuals in each subpopulation varies constantly with each iteration. Figure 4 shows a schematic diagram of GSWO with the three-population division strategy. Unlike the traditional WOA, in the block “Sorting the whale population”, Equation (12) is employed to divide the whole population into three subpopulations based on the fitness value. Approximately one-third of the individuals (exploratory subpopulation or the blue part) engage in global exploration, including Equations (7) and (8). Another third (exploitative subpopulation or the orange part) engages in local exploitation, including Equation (13), and the other third (hesitant subpopulations or the green part) inherits all traditional WOA traits, including Equation (1). Additionally, the exploratory and exploitative subpopulations implement genetic operators (the branch on the left side) in the next paragraph. After that, four branches are concatenated before calculating the fitness value and conducting the proposed conditional inherited choice. During iterative procedures, this architecture gives GSWO the flexibility to conduct both global and local explorations.

However, if only this three-population structure were used, its tendency would still be more towards exploitation than exploration for two reasons. Firstly, individuals gravitate toward random peers during the exploration phase, limiting their ability to increase exploration and expand their search field. Secondly, the WOA algorithm has two exploitation techniques: encircling prey technique and Bubble-net attack technique, while there is only one technique, searching for prey, to explore and expand the search area. A parallel branch of the genetics operator is proposed to address the problem of an imbalance between exploration and exploitation in this optimization algorithm. Exploitative and exploratory subpopulations were copied and stored in a new matrix to perform genetic operators. For this branch, the position of each whale is considered as a chromosome. Our proposed method performs a crossover between a good individual and a poor individual to create more diversity instead of a crossover between two good individuals, as in the traditional GA, which creates two new nearly identical chromosomes similar to the parents, leading to less diversity. Another point that makes our technique suitable for combination with WOA is that it only takes one child with more chromosomes from a good parent, which is the individual in exploitative subpopulations. Furthermore, to avoid bias in the exploration phase, we propose the formula presented in Equation (14) to control the number of genes changing among parents and retain some informative chromosomes. After that, we used a traditional mutation method.

(14) $$k=round(0.5\times O-0.4\times O\times \frac{t}{M}),$$

where *k* is the number of genes performing the crossover, *t* is the current iteration index, and M is the total iteration. Based on extensive experiments, it is beneficial for *k* to decrease linearly from half to 10% of the total number of genes (*O*) because, in some early iterations, individuals cannot determine which chromosomes are helpful. However, later on, individuals in the exploitative population identified that they contained chromosomes that carried much information, so fewer genes needed to be exchanged.

The number of individuals in the population increases after new individuals are created through the GA. Assuming that the WOA population is *N* and the GA population is *G*, the new population is a combined (N+G) population, leading to a population boom after some iterations. To avoid a population explosion, which leads to high computational costs, a conditional choice strategy, inspired by the Altruism strategy [44], is proposed to sacrifice individuals in the new population with the number of sacrificed whales equal to *G*. Therefore, the similarity index (SI), which is described in Equation (15), is used to calculate the similarity score of each whale with their counterparts in the population.

(15) $$SI=\frac{1}{HD\left({\vec{X}}_{1}^{t},{\vec{X}}_{2}^{t}\right)+\left|Fit_{1}^{t}-Fit_{2}^{t}\right|+\xi_{1}},$$

where ${\vec{X}}_{1}^{t},{\vec{X}}_{2}^{t}$ are candidate individuals and $Fit_{1}^{t},Fit_{2}^{t}$ are their fitness values. HD represents the Hamming distance between ${\vec{X}}_{1}^{t}$ and ${\vec{X}}_{2}^{t}$. The Hamming distance, denoted by $\left({\vec{X}}_{1}^{t},{\vec{X}}_{2}^{t}\right)$, quantifies the disparity between two binary-encoded candidate solutions by counting the points of variation. A minimal value $\xi_{1}$ is added to the denominator to prevent errors in scenarios where the denominator becomes zero. After calculating, one of the two individuals in pairs with the highest SI will be removed randomly. The number of individuals removed is equal to *G* to ensure the consistency of the algorithm, which is a final population size of *N* after each iteration.

A flowchart of the GSWO is illustrated in Figure 4. The pseudocode for the proposed method is presented in Algorithm 1.
**Algorithm 1** Pseudo-code for the GSWO
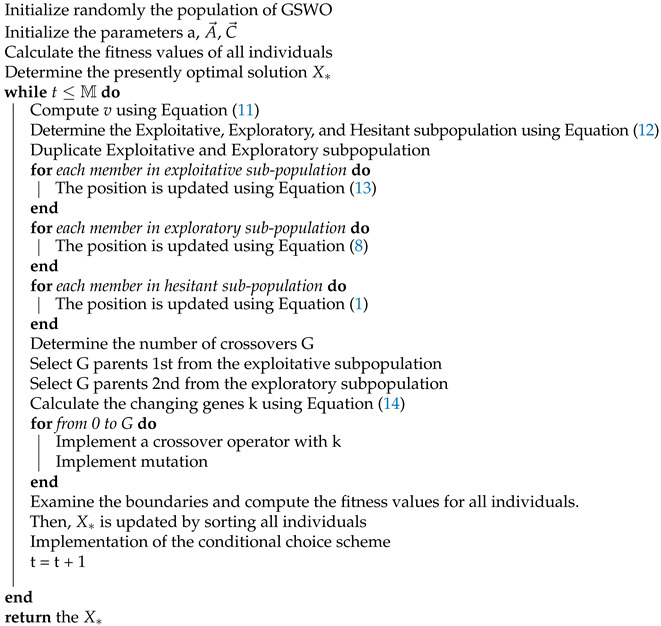


### 4.3. CatBoost Classification Model

This study employed CatBoost as a WSN data classifier and attack detector. Based on decision-tree techniques, CatBoost is a fast, high-performance gradient-boosting system. CatBoost creates balanced trees with a symmetric structure, which sets it apart from other gradient-boosting algorithms such as XGBoost and LightGBM. This implies that all the nodes in a level are subjected to the same feature-split pair that yields the lowest loss in each phase. Among the many benefits of this balanced design is its ability to build CPUs efficiently, shorten prediction times, apply models quickly, and function as a type of regularization to avoid overfitting.

CatBoost accommodates diverse data types, including numeric, categorical, and textual data. Categorical attributes, which are inherently noncomparable and pose a challenge to the direct utilization of binary trees. In contrast to prevalent gradient boosting-based methodologies such as one-hot encoding (OHE), gradient statistics (GS), target statistics (TS), greedy TS, holdout TS, and leave-one-out TS, CatBoost employs an advanced ordered TS approach. This strategy mitigates the issues related to target leakage, diminishes overfitting, and enables using a complete dataset for training purposes [39,45].

In CatBoost, the initial definition of hyperparameters is imperative for optimizing predictive accuracy. Specifically, determining parameters such as the number of trees is significant because it directly affects the potential for encountering overfitting or underfitting challenges. The parameter *d* (maximum depth) reflects the learning capacity inherent in the base tree; a higher value augments the learning process of the individual base tree, whereas a lower value diminishes it. The learning rate parameter governs the velocity at which the iterative parameters converge toward the optimal values, substantially influencing the training speed.

Previous hyperparameter optimization methods, such as random and grid search, were time-consuming but did not fully use the ranges. Thus, in this study, a GSWO approach was used to fine-tune the hyperparameters of CatBoost, and prior data were used to investigate the likely optimal parameter combination. Consequently, the GSWO-CatBoost hybrid algorithm was created.

### 4.4. Applying GSWO for Feature Selection

**Figure 5:**
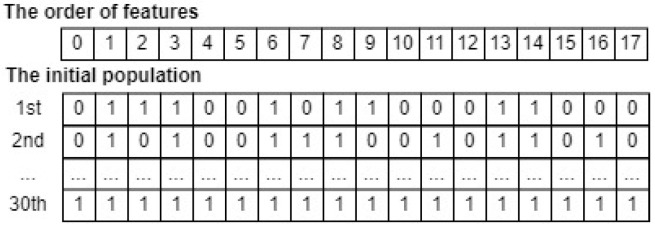
An example of an input whale population for FS.

To apply the GSWO algorithm to a specific task, defining the total iteration (M), the number of crossovers (*G*), the number of whales in the population (*N*), and the form of each whale in the population as input and a fitness function as the target for this algorithm is required. The output is the candidate whale with the lowest fitness value. In this section, we apply GSWO for FS. To begin with, the total iteration (M), the number of crossovers (*G*), and the number of feature subsets in the population (*N*) are considered to be hyperparameters, which vary based on a specific dataset. These hyperparameters must be chosen pertinently to ensure that the GSWO can achieve the most useful feature subset and optimize the computational time. In addition, the form of whales in the input population, a feature subset, is illustrated as binary arrays comprising 0s and 1s. In this representation, a position of a feature marked 0 implies exclusion, whereas a position of another feature marked 1 implies inclusion in the feature subset for calculating fitness value. Therefore, a feature subset in the input population ${\vec{X}}_{i}^{t}$ is characterized as a binary array of dimensions D, which is the number of whole features in a specific dataset. For instance, the WSN-DS dataset [46] has eighteen features. Through comprehensive experiments for this dataset, the total iteration (M), the number of crossovers (*G*), and the number of feature subsets in the population (*N*) are set as 30, 20, and 30, respectively. An initial population that has 30 feature subsets can be depicted in Figure 5. Note that an initial population is initialized randomly because the algorithm will update the feature subsets in the population to find the best subset, including only the useful features, to minimize the fitness function.

Subsequently, the fitness function for FS is defined in Equation (16), where the value of the weight β is set as 0.9 through comprehensive experiments in this study; $err({\vec{X}}_{i}^{t})$ is the false prediction rate of the CatBoost model, which is gained from training and evaluating it on a particular dataset with the features in a feature subset ${\vec{X}}_{i}^{t}$; $sum({\vec{X}}_{i}^{t})$ is the sum of features in the feature subset ${\vec{X}}_{i}^{t}$, such as $sum({\vec{X}}_{1}^{1})=8$, as depicted in Figure 5.

(16) $$Fit_{i}=\beta \times err\left({\vec{X}}_{i}^{t}\right)+\left(1-\beta \right)\times \frac{sum({\vec{X}}_{i}^{t})}{D}.$$

The fitness function is a weighted sum of the classification model error and the proportion of features present in the candidate solution. Therefore, the optimization goal involves minimizing the fitness function. This fitness function ensures a reduction in the rate of false predictions and that as few features as possible are maintained.

**Figure 6:**
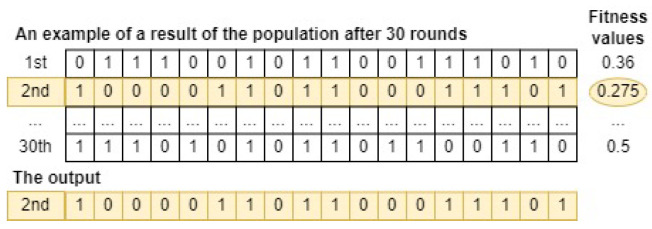
An example of a result of GSWO for FS.

Finally, Algorithm 1 is applied to run the GSWO algorithm 30 rounds. As a result, a feature subset with the lowest fitness value, corresponding to the most useful features subset, is determined. Figure 6 illustrates that the second feature subset, which has the lowest fitness value, is chosen as the output of GSWO for FS. In summary, the most useful feature subset, represented by the second whale, consists of features of order [0, 5, 6, 8, 9, 13, 14, 15, 17].

### 4.5. Applying GSWO for Hyperparameter Optimization

This study developed an ensemble-learning approach based on CatBoost to identify intrusive behaviors in WSNs. The optimization of CatBoost hyperparameters poses a challenge, promoting the proposal of GSWO and quantization [47] are introduced as solutions. The hyperparameters considered in this study include learning rate (α), depth (d), iteration (iter), L2 regularization term (l2), bagging_temperature (b), and random_strength (r). The roles of these parameters in CatBoost’s performance are as follows:

CatBoost typically employs 1000 trees by default. Consequently, a higher number of iterations leads to increased training and inference times, while excessively few iterations may yield subpar results. Striking an optimal balance in the number of iterations is crucial for maintaining speed and accuracy. Simultaneously, adjusting the learning rate became necessary as the number of iterations decreased, emphasizing the importance of selecting an appropriate learning rate for optimal model performance. Although CatBoost’s default learning rate is often close to optimal, it can be finetuned to achieve the best-suited values. Similarly, hyperparameters such as depth, random strength, bagging temperature, and L2 regularization terms require optimization to identify the tradeoff that maximizes accuracy.

To apply GSWO for hyperparameter optimization, the form of each whale in the input population must be a binary array. However, the types of these hyperparameters, integer or float, are unsuitable for the input of our proposed method GSWO and do not optimize the computing resources regarding speed and accuracy. Therefore, we proposed quantization to convert these hyperparameters into a binary array before feed-forwarding to GSWO. The quantization included three steps:

Step 1: Calculate the range of values within the specified range using Equation (17).

(17) value_range=max_value−min_value.

Step 2: Calculate the step size for each bit using Equation (18), where num_bits is the total number of bits used for representation.

(18) $$step\_size=\frac{value\_range}{2^{num\_bits}}.$$

Step 3: Quantize the number using Equation (19) and convert it to a binary array.

(19) $$quantized\_value=round\left(\frac{number-min\_value}{step\_size}\right),$$

where the number is the value to be quantized.

Each hyperparameter is represented by the number of bits appropriate to its allowable value range. In this study, through extensive experiments, the specific range of each hyperparameter and the number of bits is set as follows:

- $\alpha \in range\left[0.001,0.5\right]$ is illustrated by 14 bits.
- $d\in range\left[2,9\right]$ is illustrated by 3 bits.
- $iter\in range\left[50,690\right]$ is illustrated by 6 bits.
- $l2\in range\left[2,9\right]$ is illustrated by 3 bits.
- $r\in range\left[0,10\right]$ is illustrated by 8 bits.
- $b\in range\left[0,10\right]$ is illustrated by 8 bits

**Figure 7:**
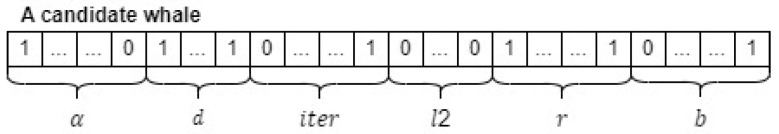
An example of a candidate whale for hyperparameter optimization.

**Figure 8:**
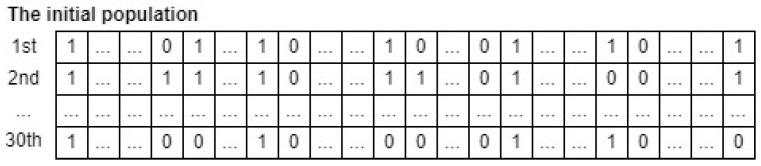
An example of an input whale population for hyperparameter optimization.

As a result, the form of a candidate whale in the input population is illustrated in Figure 7. Through comprehensive experiments for fine-tuning hyperparameters of the CatBoost model, the total iteration (M), the number of crossovers (*G*), the number of whales in the population (*N*) are set as 40, 20, and 30, respectively. In addition, an initial population of 30 whales can be depicted in Figure 8. Note that an initial population is initialized randomly.

The fitness function for hyperparameter optimization was different from that for FS. To optimize hyperparameters to enhance the accuracy and inference speed, minimizing the error of the classification model, iterations, and depth is necessary. Therefore, the fitness function for hyperparameter optimization, which is the weighted sum of the errors of the classification model, iterations, and depth, is defined by Equation (20). Therefore, the optimization goal involves minimizing the fitness function.

(20) $$Fit_{i}=\gamma \times err\left({\vec{X}}_{i}^{t}\right)+\frac{1-\gamma }{2}\times iter+\frac{1-\gamma }{2}\times d,$$

where the value of the weight γ is set as 0.9 through comprehensive experiments in this study; $err({\vec{X}}_{i}^{t})$ is the false prediction rate of the CatBoost model, which is gained from training and evaluating it on a specific dataset with the hyperparameters established in the candidate whale ${\vec{X}}_{i}^{t}$.

**Figure 9:**
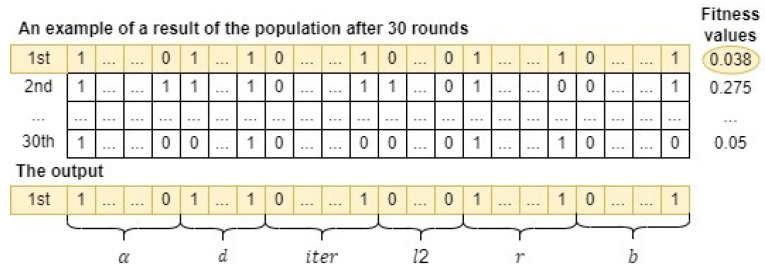
An example of a result of GSWO for hyperparameter optimization.

Finally, Algorithm 1 is applied to run the GSWO algorithm 30 rounds. As a result, a candidate whale with the lowest fitness value, corresponding to the most effective hyperparameters for the CatBoost model to process a specific dataset, is determined. Figure 9 illustrates that the first whale, which has the lowest fitness value, is chosen as the output of GSWO for hyperparameter optimization.

## 5. Experiments and Evaluations

### 5.1. Dataset Description

#### 5.1.1. NSL-KDD Dataset

The first dataset, NSL-KDD from [46], is a typical dataset used for classification during implementation. This dataset has 41 unique parameters, including the content type, core category, and traffic categorization, which are thoroughly explained in Table 2. Each data input reveals a wide variety of flow characteristics, each of which is methodically classified as an attack category or a normal instance.

Furthermore, the 42nd presents five unique types of system connection vectors. These vectors are then classified into a normal category and four distinct attack types. As outlined in [49], the four categories of attacks—Denial of Service (DoS), Probe, Remote to Local (R2L), and User to Root (U2R)—are further categorized into specific types of intrusions.

This dataset is considered a merging set, which is divided into training and testing sets with a rate of 7:3. The statistics of each attack class are listed in Table 3. We used this dataset to demonstrate that our method works well for both categorical features and numerical features.

#### 5.1.2. CICIDS2017 Dataset

The CICIDS-2017 dataset, sourced from the ISCX Consortium, has a prominent position in the landscape of WSN research, particularly in the domain of network intrusion detection. This dataset is a valuable resource for investigating and understanding network security challenges in real-world contexts. With its systematic arrangement, the dataset encompasses eight discrete traffic monitoring sessions, each presented in a comma-separated value (CSV) format. The categorization of network traffic involves the classification into “Benign” for regular activities and “Attacks” for a diverse array of intrusion attempts. Offering a rich array of 14 different attack types, including sophisticated scenarios such as Infiltration, Bot attacks, and DDoS, the dataset enables researchers to explore complex attack patterns reflective of contemporary network environments.

According to CICIDS-2017, datasets characterized by a notable class imbalance tend to exhibit diminished detection accuracy and increased false alarm rates. The approach advocated by Karimi et al. [50] and Panigrahi and Borah [51] proposed novel labeling methodologies for attack traffic, as shown in Table 4. Additionally, because many instances of normal traffic were included in this dataset, we extracted only 20% of the normal traffic data for training and testing. The numbers of each class are also shown in Table 4.

Moreover, including advanced features tailored to detect specific attack types enhances their utility in modeling and analyzing the intricacies of network intrusions. Thus, the CICIDS-2017 dataset is a crucial tool for conducting in-depth research and evaluating the intrusion detection model [52]. This dataset was also separated into two sets for training and testing, the distribution of which is presented in Table 3.

#### 5.1.3. WSN-DS Dataset

A Wireless Sensor Network Dataset (WSN-DS) [21] facilitates the application of diverse intelligent and data mining methodologies to enhance the detection and classification of Denial of Service (DoS) attacks. This leads to sensor nodes acquiring proficiency in discerning normal behaviors and promptly identifying the signatures of attackers, thereby enabling timely and well-informed decision-making. The dataset employed the LEACH routing protocol to extract 23 attributes crucial for evaluating the status of each node within the network. It is noteworthy that, in the CSV files, certain attributes such as RSSI, max distance to CH, the average distance to CH, and current energy are removed.

Within this dataset, researchers have specifically delineated four types of DoS attacks using the LEACH protocol. These encompass Blackhole, Grayhole, Flooding, and Scheduling (or TDMA) attacks. Notably, the dataset includes a category for “Normal” nodes, serving as a reference for non-attacker nodes. This initiative ensures that the dataset comprehensively covers both malicious and benign network behaviors, thereby fostering the development and evaluation of robust intrusion detection mechanisms tailored to WSNs. The distributions of 70% of the dataset for training and 30% for testing are presented in Table 3.

#### 5.1.4. WSN-BFSF Dataset

The WSN-BFSF dataset incorporates instances of Blackhole, Flooding, and Selective Forwarding attacks. Unlike the WSN-DS dataset, the WSN-BFSF dataset encompassed data specifically related to the Selective Forwarding attack. This dataset, comprising 312,106 instances across 16 distinct features, presents a comprehensive landscape for the analysis of WSN traffic, particularly focusing on the identification and differentiation of various attack vectors along with normal network operations. The dataset encapsulates a diverse range of traffic types, including Blackhole attacks, Flooding attacks, Selective Forwarding attacks, and normal traffic. This rich compilation of data, derived from post-meticulous preprocessing, provides an invaluable resource for deepening our understanding of network vulnerabilities and the dynamics of cyber threats in WSNs.

### 5.2. Evaluation Metrics

In this investigation, widely utilized metrics in academic literature, including accuracy, precision, recall, F-score, inference time, and ROC curves, were employed to evaluate the performance results. These metrics are applicable in diverse classification scenarios [53,54]. The assessment values are derived by comparing the classification outcomes generated by various ML or deep learning algorithms with the specified classification values, thereby effectively interpreting the model outputs. These metrics rely on the foundation of a confusion matrix incorporating key elements such as true positives (TPs), true negatives (TNs), false positives (FPs), and false negatives (FNs). TP signifies cases correctly identified as attacks, TN represents samples accurately categorized as normal, FP indicates normal samples misclassified as attack samples, and FN denotes attack samples erroneously classified as normal samples.

Equation (21) formulates the accuracy parameter as the ratio of all correctly classified samples (TP, TN) to the total samples (TP, TN, FP, and FN). Precision, as defined in Equation (22), quantifies the number of accurate positive (TP) predictions, emphasizing the ratio of all correctly classified attacks (TP) to the total number of correctly classified attacks (TP) and erroneously classified normal samples (FP). Equation (23) computes recall as the number of accurate positive predictions out of all potential positive predictions. Precision considers only accurate positive predictions, whereas recall encompasses both accurate and inaccurate predictions. The F1-score (Equation (24)) serves as a balanced measure of model performance, relying on the harmonic mean of sensitivity and recall. The F1-score, which assigns equal importance to precision and recall, has emerged as a frequently utilized metric in the realm of learning from data [55]. Inference time, which represents the time it takes the model to infer the entire test set, was also incorporated as a metric for comparison with other studies. Additionally, a receiver operating characteristic (ROC) curve was used to plot the true positive rate against the false positive rate for different threshold values. In ROC curves, the *x*-axis shows the false positive ratio, whereas the *y*-axis shows the true positive ratio. Therefore, results close to the upper-left corner were considered ideal. Moreover, a ten-fold cross-validation approach was implemented for each model in our experiments to ensure realistic and dependable results.

(21) $$Accuracy=\frac{TP+TN}{TP+TN+FP+FN}.$$

(22) $$Precision=\frac{TP}{TP+FP}.$$

(23) $$Recall=\frac{TP}{TP+FN}.$$

(24) $$F1-score=\frac{2*Precision*Recall}{Precision+Recall}.$$

### 5.3. Results and Analysis

In this section, the efficacy of the proposed model is assessed. All experimental trials were conducted on a Windows 11 personal computer equipped with an Intel Core i7 CPU operating at 3.80 GHz and with 16 GB of RAM.

**Figure 10:**
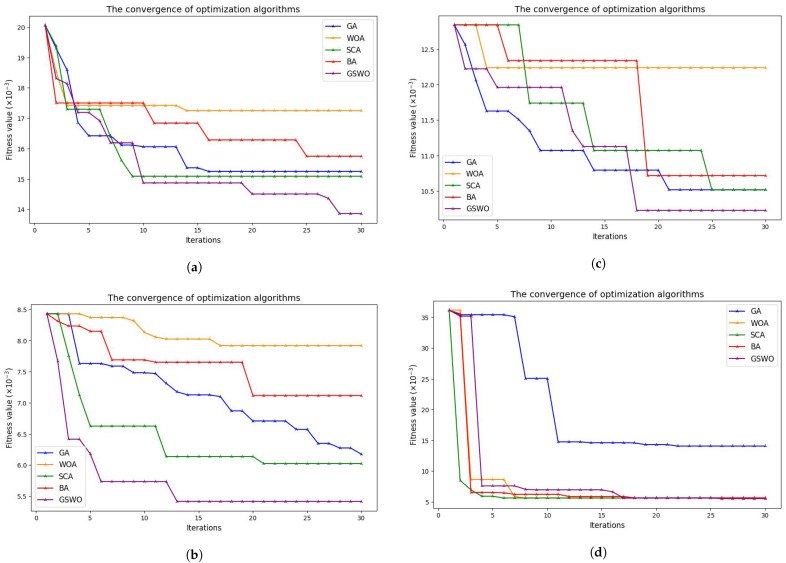
The convergence of algorithms for (**a**) NSL−KDD, (**b**) CICIDS2017, (**c**) WSN−DS, and (**d**) WSNBFSF.

In this study, we selected four typical metaheuristic algorithms, namely the original WOA, the original GA, sine–cosine algorithm (SCA), and bat algorithm (BA), for comparison with our proposed GSWO algorithm in terms of the level of convergence and ability to select useful features. Figure 10 show the level of convergence of the algorithms during the FS process, Table 5 illustrates the features selected from the algorithms, and Table 6 compares the performance using classification metrics. These algorithms set the number of populations to 30 and the maximum iteration to 30. In this comparison, hyperparameters are set as follows: iterations: 500; learning_rate: 0.05; depth: 2; l2_leaf_reg: 3.0; random_Strength: 1; bagging_templates: 1; od_wait: 20; the remaining are the default. Note that the highest results are shown in boldface.

The findings are illustrated in Figure 10, which emanate from a comparative analysis involving the proposed GSWO algorithm and other prominent algorithms. GSWO takes advantage of the good exploitation capabilities of the conventional WOA, boosts global search capabilities through the parallel use of extra genetic operators, and strikes a balance between exploratory and exploitative tendencies. Especially noteworthy is its performance in the context of large datasets, demonstrating superior outcomes as shown in Figure 10b for the CICIDS2017 dataset. These empirical observations substantiate the GSWO’s efficacy in circumventing local optima, establishing its capacity to find optimal solutions with greater precision than alternative algorithms, particularly the traditional WOA.

The results, which are illustrated in Table 6, prove that the features selected by the proposed GSWO improved the classification rate on various datasets in terms of all classification metrics compared with the traditional WOA, GA, and other metaheuristic optimizations and raw data. In particular, the results of the proposed method on the CICIDS 2017 dataset demonstrate that it can work effectively with a large dataset.

To evaluate the performance of our proposed algorithm for hyperparameter optimization, we used some of the most popular hyperparameter tuning methods, such as random search, grid search, and Optuna. Table 7 presents the values of CatBoost’s hyperparameters, which are the results of the algorithms, and Table 8 compares the efficiencies of these methods based on the evaluation metrics as presented in Section 5.2. The useful features selected from the GSWO-based FS were used in this comparison.

A detailed comparative analysis of the metrics was used to evaluate the performance of some popular hyperparameter optimization approaches, such as grid search, random search, and Optuna, and the proposed method is presented in Table 8. After the experiments, we conclude that the proposed method can find productive hyperparameters to assist the classifier in correctly detecting and classifying various types of attacks. Therefore, the results indicate an enhancement of the proposed model for solving intrusion detection problems in complex and diverse WSN environments.

**Figure 11:**
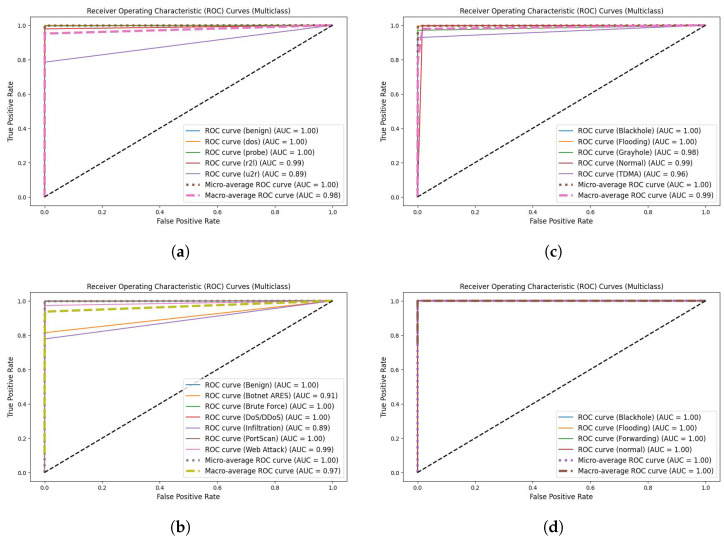
ROC curves for proposed system in (**a**) NSL-KDD dataset, (**b**) CICIDS2017 dataset, (**c**) WSN-DS dataset, and (**d**) WSNBFSF dataset.

In addition, from the ROC curves shown in Figure 11, the proposed algorithm was quite successful for all classes in the four different datasets.

Furthermore, to ensure realistic and reliable results, a ten-fold cross-validation approach was implemented for each dataset in our experiments, as illustrated in Table 9.

Finally, Table 10 shows a comparison of the performance of the proposed system with state-of-the-art techniques for each dataset in WSNs. As shown in Table 10, the proposed system surpasses other existing methods in the WSN field in terms of accuracy, precision, recall, F1-score, and inference time. This approach leverages a GSWO-based technique for FS in sensor-node traffic data, effectively mitigating the dimensionality of traffic features. Subsequently, a CatBoost algorithm coupled with GSWO-based hyperparameter optimization was employed to enhance the accuracy of the model, uphold a commendable detection rate, and reduce computational time overhead. This method addresses the prevalent shortcomings of existing FS and classification algorithms in WSN IDSs, notably the issues of suboptimal detection performance and limited real-time responsiveness. The proposed model demonstrated robust detection capabilities and superior real-time performance.

## 6. Conclusions

In conclusion, this research marks a significant advancement in the intrusion detection domain of WSNs. The proposed GSWO combines GA and WOA, modified by applying a three-population division strategy with conditional inherited choice (CIC). This approach addresses the critical demand for effective feature selection (FS) in WSN security, overcoming the limitations of conventional methods, such as premature convergence. GSWO, which ingeniously combines a genetic algorithm (GA) and the modified whale optimization algorithm (WOA) with a conditional inherited choice (CIC), not only achieves a balance between exploration and exploitation but also enhances global search abilities. In addition, by leveraging the advanced capabilities of the CatBoost model as a classifier and introducing a new technique based on GSWO and the quantization for fine-tuning CatBoost’s hyperparameters, our system demonstrated efficiency in WSN intrusion detection. The comprehensive evaluations across diverse datasets, including both benchmarks and specialized WSN datasets, demonstrate the accuracy and real-time applicability of our method. Specifically, our method outperformed existing methods with impressive accuracy rates of 99.65% for WSN-DS, 99.99% for WSNBFSF, 99.76% for NSL-KDD, and 99.74% for CICIDS2017. These results underscore the effectiveness of our approach in enhancing the accuracy of intrusion detection systems in WSNs. Furthermore, our GSWO-CatBoost method significantly reduces the inference time, making it nearly 100 times faster than deep learning methods. This notable reduction in inference time is a critical advancement in meeting the ever-increasing demand for real-time applications in WSN intrusion detection.

Our future work will aim to optimize the modified WOA further by integrating filter-based FS methods, particularly by exploring the information gained. This iterative refinement aims to enhance the ability of the algorithm to select relevant features, contributing to its overall intrusion detection performance.

## Figures and Tables

**Table 1:** Some recent works about intrusion detection in WSN.

Year	Authors	Feature Selection	Model	Parameters Fine-Tuning	Type of Classification	Dataset	Accuracy (%)
2023	Salmi and Oughdir [26]	None	DNN, CNN, RNN, CNN+RNN	None	Multi-class	WSN-DS	97.04, 98.79, 96.48, 96.86
2020	Jiang et al. [27]	SBS	LightGBM	None	Multi-class	WSN-DS	99.53
2019	Vinayakumar et al. [23]	None	DNN	None	Multi-class & Binary	KDD Cup’99, NSL-KDD, UNSW-NB15, CICIDS2017, WSN-DS	95.00–99.00, 95.00–99.00, 65.00–75.00, 93.00–96.00, 96.00–99.00
2018	Le et al. [6]	None	Random Forest	None	Multi-class	WSN-DS	98.00
2021	Liu et al. [28]	PSO-LightGBM	OCSVM	None	Multi-class	UNSW-NB15	86.68
2020	Vijayanand et al. [29]	WOA-GA (sequentially)	SVM	None	Multi-class	CICIDS2017, ADFA-LD	95.91, 94.44
2023	Mohiuddin et al. [31]	WOA-SCA	XgBoost	None	Multi-class & Binary	UNSW-NB15, CICIDS2017	91.00–99.00, 96.00–98.00
2023	Kasongo et al. [32]	GA	RF	None	Multi-class	UNSW-NB15	87.61
2022	Hussain et al. [30]	WOA-ABC	DNN	None	Multi-class	NSL-KDD	98.00
2024	Our	GSWO	CatBoost	GSWO	Multiclass	NSL-KDD, CICIDS2017, WSN-DS, WSNBFSF	99.79, 99.74, 99.62, 99.99

**Table 2:** NSL-KDD Dataset description [48].

Criteria	Description
Dataset’s name	NSL-KDD
Quantity of records	149,470
Quantity of network features	41
Quantity of attack categories	4 (DoS, Probe, R2L, U2R)
Characteristics of network features	Basic features, host features, traffic features, and content features

**Table 3:** Summarizing the distribution of training and testing set in this study.

Datasets	The Traffic Type	Training Set	Testing Set
NSL-KDD dataset	Benign	54,093	22,960
	Dos	37,424	16,138
	Probe	9780	4299
	R2L	2490	1079
	U2R	173	79
CICIDS2017 dataset	Normal	260,415	65,266
	Bot	1561	382
	Brute Force	6824	1727
	DoS/DDoS	256,401	63,868
	Infiltration	27	9
	Portscan	45,779	11,526
	Web Attack	1716	402
WSN-DS dataset	Blackhole	8104	1945
	Grayhole	10,598	2624
	Flooding	2405	597
	TDMA	5306	1322
	Normal	259,621	65,020
WSN-BFSF dataset	Normal	210,223	52,628
	Flooding	23,913	5931
	Blackhole	9441	2325
	Forwarding	6108	1537

**Table 4:** Data distribution of relabeled attack.

New Labels	Old Labels	Distribution	Percentage
Normal	Benign	325,681	45.49
Bot	Bot	1943	0.27
Brute Force	FTP-Patator, SSH-Patator	8551	1.19
DoS/DdoS	DDoS, DoS Hulk, Heartbleed, Slow, DoS slowloris, GoldenEye, DoS, httptest	320,269	44.74
Infiltration	Infiltration	36	0.005
Portscan	Portscan	57,305	8.01
Web Attack	Web Attack-Sql Injection, Web Attack-XSS, Web Attack-Brute Force	2118	

**Table 5:** Features selected after FS technology.

Datasets	Feature Selection Methods	Feature Orders
WSN-DS	WOA	[0, 5, 6, 7, 8, 9, 13, 14, 15, 17]
	GA	[3, 5, 6, 8, 9, 11, 12, 14, 15, 17]
	SCA	[2, 3, 5, 8, 9, 11, 14, 17]
	BA	[0, 4, 5, 6, 8, 9, 13, 14, 15, 17]
	Our GSWO	[0, 5, 6, 8, 9, 13, 14, 15, 17]
WSNBFSF	WOA	[0, 1, 3, 4, 7, 9, 10, 12]
	GA	[0, 1, 2, 3, 4, 5, 6, 7, 9, 10, 12]
	SCA	[3, 4, 7, 8, 10]
	BA	[0 2 4 6 8 14]
	Our GSWO	[0, 3, 4, 10, 12]
NSL-KDD	WOA	[0, 2, 4, 5, 7, 13, 14, 15, 17, 18, 21, 22, 23, 25, 26, 27, 29, 32, 34, 36, 37, 38, 41]
	GA	[0, 2, 4, 5, 9, 12, 22, 23, 26, 27, 29, 31, 32, 33, 35, 36, 37, 39, 40, 41]
	SCA	[2, 3, 4, 5, 19, 20, 22, 28, 29, 31, 35, 36, 38, 41]
	BA	[0, 2, 4, 5, 9, 12, 16, 22, 23, 25, 27, 29, 31, 33, 35, 36, 39, 40, 41]
	Our GSWO	[0, 1, 2, 4, 15, 21, 22, 23, 26, 27, 31, 32, 33, 34, 35, 36, 38, 40, 41]
CICIDS2017	WOA	[0, 1, 3, 5, 6, 14, 16, 17, 18, 19, 20, 26, 28, 33, 34, 37, 40, 43, 45, 54, 55, 56, 58, 59, 61, 62, 66]
	GA	[0, 8, 12, 13, 15, 17, 19, 20, 24, 26, 30, 32, 34, 38, 39, 44, 48, 55, 56, 58, 59, 60, 62]
	SCA	[0, 15, 16, 24, 38, 41, 42, 48, 54, 55, 56, 62]
	BA	[0, 3, 4, 7, 10, 11, 12, 13, 19, 22, 23, 24, 27, 32, 33, 41, 42, 46, 51, 52, 55, 56, 58, 62, 65]
	Our GSWO	[0, 1, 8, 10, 13, 24, 25, 31, 34, 41, 42, 44, 57, 65]

**Table 6:** Comparison of performance metrics of FS algorithms.

Datasets	Method	Acc	Prec	Rec	F1
WSN-DS	All features	98.01	92.81	85.80	89.01
	WOA	98.23	93.53	**88.87**	90.90
	GA	98.24	93.58	88.86	90.92
	SCA	98.23	93.49	88.86	90.88
	BA	98.23	93.50	**88.87**	90.88
	Our GSWO	**98.25**	**93.62**	88.85	**90.93**
WSNBFSF	All features	93.81	46.97	50.00	48.44
	WOA	96.76	94.78	76.74	82.91
	GA	96.76	94.66	77.74	82.89
	SCA	99.13	96.43	95.95	96.18
	BA	99.58	98.53	98.39	98.45
	Our GSWO	**99.68**	**98.64**	**98.92**	**98.77**
NSL-KDD	All features	98.40	96.29	77.10	77.78
	WOA	98.49	91.44	80.89	82.92
	GA	98.87	**95.47**	85.94	88.44
	SCA	98.42	93.62	78.87	79.97
	BA	98.86	95.34	81.63	83.31
	Our GSWO	**98.89**	94.33	**86.92**	**89.19**
CICIDS2017	All features	95.23	40.45	41.60	41.00
	WOA	99.00	69.50	67.74	68.52
	GA	99.13	69.17	68.58	68.84
	SCA	99.35	82.54	77.83	79.81
	BA	99.01	68.85	68.52	68.66
	Our GSWO	**99.37**	**98.35**	**81.90**	**86.22**

**Table 7:** Results of hyperparameter optimization techniques.

Datasets	Techniques	Hyperparameter Values
WSN-DS	Grid search	[‘iter’: 211, ‘α’: 0.1, ‘d’: 10, ‘l2’: 1, ‘r’: 1, ‘b’: 1]
	Random search	[‘iter’: 204, ‘α’: 0.1, ‘d’: 10, ‘l2’: 3, ‘r’: 2, ‘b’: 2]
	Optuna	[‘iter’: 427, ‘α’: 0.46122, ‘d’: 11, ‘l2’: 2.5, ‘r’: 1, ‘b’: 2.748]
	Our method	[’iter’: 370, ‘α’: 0.3033, ‘d’: 6, ‘l2’: 2.0, ‘r’: 8.9063, ‘b’: 0.7031]
WSNBFSF	Grid search	[‘iter’: 179, ‘α’: 0.1, ‘d’: 10, ‘l2’: 1, ‘r’: 4.5, ‘b’: 2]
	Random search	[‘iter’: 216, ‘α’: 0.07, ‘d’: 10, ‘l2’: 3, ‘r’: 1, ‘b’: 1]
	Optuna	[‘iter’: 912, ‘α’: 0.3768, ‘d’: 10, ‘l2’: 6, ‘r’: 1, ‘b’: 7.577]
	Our method	[‘iter’: 270, ‘α’: 0.28817, ‘d’: 5, ‘l2’: 2.0, ‘r’: 0.15625, ‘b’: 1.09375]
NSL-KDD	Grid search	[‘iter’: 235, ‘α’: 0.1, ‘d’: 10, ‘l2’: 1, ‘r’: 5, ‘b’: 5]
	Random search	[‘iter’: 284, ‘α’: 0.1, ‘d’: 10, ‘l2’: 3, ‘r’: 2, ‘b’: 2]
	Optuna	[‘iter’: 515, ‘α’: 0.28, ‘d’: 6, ‘l2’: 2, ‘r’: 8.95, ‘b’: 3.34]
	Our method	[‘iter’: 515, ‘α’: 0.3, ‘d’: 6, ‘l2’: 2, ‘r’: 8.9, ‘b’: 3.3]
CICIDS2017	Grid search	[‘iter’: 220, ‘α’: 0.1, ‘d’: 10, ‘l2’: 3, ‘r’: 2.5, ‘b’: 3]
	Random search	[‘iter’: 220, ‘α’: 0.1, ‘d’: 10, ‘l2’: 3, ‘r’: 2.5, ‘b’: 3]
	Optuna	[‘iter’: 376, ‘α’: 0.3246, ‘d’: 10, ‘l2’: 5, ‘r’: 1, ‘b’: 8.31]
	Our method	[‘iter’: 194, ‘α’: 0.2783, ‘d’: 9, ‘l2’: 2, ‘r’: 7.707, ‘b’: 2]

**Table 8:** Comparison of performance metrics of hyperparameter optimization techniques.

Dataset	Method	Acc	Prec	Rec	F1
WSN-DS	Grid search	**99.62**	97.31	97.46	97.42
	Random search	99.59	97.18	97.69	97.37
	Optuna	99.60	**97.39**	97.51	97.40
	Our method	**99.62**	97.27	**97.78**	**97.47**
WSNBFSF	Grid search	99.98	99.98	99.97	99.98
	Random search	99.98	99.96	99.96	99.96
	Optuna	99.97	99.94	99.82	99.88
	Our method	**99.99**	**99.99**	**99.99**	**99.99**
NSL-KDD	Grid search	97.66	95.33	93.76	94.89
	Random search	99.76	96.37	93.99	95.05
	Optuna	99.75	**96.77**	95.71	96.21
	Our method	**99.79**	96.46	**96.48**	**96.47**
CICIDS2017	Grid search	99.73	97.68	89.67	92.81
	Random search	99.73	**98.05**	87.89	91.32
	Optuna	99.70	97.66	90.02	92.88
	Our method	**99.74**	97.39	**93.68**	**95.32**

**Table 9:** Validation of the proposed method with the 10-fold cross-validation technique.

Dataset	Detection Rate (%)
WSN-DS	99.62 ± 00.04
WSNBFSF	99.99 ± 00.00
NSL-KDD	99.82 ± 00.03
CICIDS2017	99.76 ± 00.01

**Table 10:** Comparison of performance metrics of existing ML techniques.

Datasets	Method	Acc	Prec	Rec	F1	Infer Time
WSN-DS	CNN [26]	98.75	95.03	92.45	93.60	5.62 s
	DNN [23]	96.40	97.00	96.40	96.60	2.90 s
	Our method	**99.65**	**97.27**	**97.78**	**97.47**	**16 ms**
WSNBFSF	LSTM-CNN [22]	95.75	95.39	95.75	95.52	6.56 s
	GRU [22]	99.01	99.00	99.01	98.99	5.91 s
	Our method	**99.99**	**99.99**	**99.99**	**99.99**	**13 ms**
NSL-KDD	DNN [23]	94.14	88.76	88.67	88.48	1.87 s
	AE [56]	89.82	91.81	90.16	90.98	3.51 s
	Our method	**99.76**	**96.17**	**95.14**	**95.63**	**37 ms**
CICIDS2017	DNN [23]	95.60	96.20	92.60	94.70	5.76 s
	CNN [26]	98.62	93.20	78.34	81.62	25.36 s
	Our method	**99.74**	**97.39**	**93.68**	**95.32**	**73 ms**

## Data Availability

Data are contained within the article (Section 5.1).
